# Sunlight exposure does not influence ICU survival

**DOI:** 10.1186/cc9954

**Published:** 2011-03-11

**Authors:** R Castro, S Hong, C Lee, L Weissfeld, G Clermont, D Angus, M Rosengart

**Affiliations:** 1 University of Pittsburgh Medical Center, Pittsburgh, PA, USA

## Introduction

The biological influence of light on human physiology (for example, circadian rhythms, cortisol and melatonin) has long been recognized. Recent interest has been directed to understanding the ramifications of light on immune function, particularly in the context of patient care. Although light and seasonal variations have been shown to modulate leucocyte count and lymphocytes B and T activity and proliferation in mammals, little is understood about these interactions in the course of illness. Thus, we hypothesized that sunlight is directly associated with improved outcome in critically ill patients.

## Methods

We conducted a retrospective, cohort study of all patients admitted to the ICU at a single center between the years 2000 and 2004. Light exposure was assessed by theoretical insolation (kWh/m^2^/day), a measure of solar energy striking a unit of earth surface area that was obtained from the National Aeronautics and Space Administration. Daily and total insolation was determined for each patient, accounting for hospital geographic location, period and duration of hospitalization, and adjusted by day-specific admission/discharge-sunrise/sunset times. To adjust for differences in case mix, we abstracted data regarding patient age, race, injury severity score, length of stay (LOS) and admission diagnostic categories. Patients who died before 24 hours were excluded from the analysis. The hypothesis was modeled using a multivariate logistic regression submodel for survival and a linear mixed submodel for the insolation measurement. Both were linked by the random intercept parameter in the mixed submodel.

## Results

A total of 22,730 patients were available for study. The majority was male (52.7%) and Caucasian (80.0%) with a mean age of 59.4 ± 17.7 years. The leading ICU admission diagnoses were cardiovascular conditions (23.4%), trauma (13.4%) and sepsis (5.9%). The mean APACHE III score was 52.8 ± 28.3, LOS was 3.3 ± 2.3 days and mortality was 13.4%. The total insolation per patient was 14.8 ± 33.2 kWh/m^2 ^(mean ± SD). After adjusting for differences in case mix, there was no significant association between survival and total insolation dose at 24 hours and up to the 10th day of ICU hospitalization (*P *= 0.64). A tendency towards lower mortality with higher insolation was observed for trauma patients (*P *= 0.15), although this did not attain statistical significance.

## Conclusions

Sunlight has no impact on general ICU patient survival according to our analysis. In other relevant outcomes (mechanical ventilation requirements, sedation, delirium incidence, and so forth), the impact of sunlight still has to be elucidated.

